# Adherence to diabetic self-care management and associated factors among type 2 diabetic patients in North Shewa Zone public hospitals in Amhara Region, Ethiopia

**DOI:** 10.3389/fcdhc.2025.1560907

**Published:** 2025-05-09

**Authors:** Agizew Endale, Fitsum Hundessa, Eyasu Tamru, Fetene Nigussie, Minyahl Hailu

**Affiliations:** ^1^ Department of Nursing, Debre Berhan Health Science College, Debre Berhan, Ethiopia; ^2^ Department of Midwifery, School of Nursing and Midwifery, Debre Berhan University, Debre Berhan, Ethiopia; ^3^ Department of Nursing, School of Nursing and Midwifery, Debre Berhan University, Debre Berhan, Ethiopia; ^4^ Department of Midwifery, Debre Berhan Health Science College, Debre Berhan, Ethiopia

**Keywords:** type 2 diabetes mellitus, adherence to diabetes self-care practice, factors, North Shewa Zone, Ethiopia

## Abstract

**Introduction:**

Adherence to diabetes self-care management is a lifestyle modification for people with diabetes.

**Objective:**

To assess adherence to diabetic self-care management and associated factors among type 2 diabetic patients in North Shewa Zone public hospitals, Ethiopia, 2023.

**Methods:**

The study employed a concurrent mixed-methods approach among 600 type 2 diabetic patients in North Shewa Zone public hospitals in Amhara, Ethiopia. The study was conducted from May 5 to May 20, 2023. The quantitative data were collected by using a semi-structured interview-administered questionnaire and chart review. Logistic regression was employed, and a p-value < 0.05 was considered statistically significant. Qualitative data were collected by in-depth interviews, and audio recordings were first transcribed verbatim and then translated to the English language by the first author and analyzed manually using a thematic approach.

**Result:**

Out of the total 600 type 2 diabetic patients, 262 (43.7%) with 95% CI: 40–47.8% of the study participants had good adherence to diabetes self-care practices. The multivariable analysis indicated that type 2 diabetic patients who lived in urban areas [AOR: 5.4, 95% CI: (1.05-8.7)] were 5.4 times more likely to have good diabetic self-care practice than those rural residents. Those who had a high school level of education [AOR: 2.9, 95% CI: (1.3-6.6)] were 2.9 times more likely to have good self-care practice, and those with college and above [AOR: 5, 95% CI (2–12):] were five times more likely to have good self-care practice. Regarding occupation, unemployed people were 66% less likely to have good self-care practices than employed people. Those who had no availability of healthcare services [AOR: 0.19, 95% CI: (0.09-0.37)] were less likely by 81% to have good self-care practice than those who had availability of healthcare services. These are significantly associated with diabetic self-care practice. The qualitative component clarified six themes: lack of education and awareness, financial affordability, accessibility, lack of family support, and having diabetic-related complications were identified as barriers.

**Conclusion:**

This study indicated that adherence of patients with type 2 diabetes to the recommended self-care practices was considerably poor. Different factors included the respondents who had a high school level or higher level of education and those who lived in urban areas. This was supported by the results from the qualitative part and thus the endorsement to strengthen diabetes health education to patients and their families. So, diabetic patients require an integrated approach through treatment as well as health education, which will increase the health and well-being of the patient.

## Background

According to estimations from the World Health Organization (WHO), type II diabetes mellitus (T2DM) is the third-highest risk factor for early mortality globally, behind only high blood pressure and cigarette use. Furthermore, considerable epidemiologic research demonstrates that T2DM incidence is rising globally. It affects 463 million people and is expected to affect 629 million people by the year 2045, making up around 90% of all diabetic patients (1). Effective management of T2D requires individuals to adhere to a comprehensive self-care regimen, which includes lifestyle modifications such as dietary changes, regular physical activity, blood glucose monitoring, medication adherence, and routine medical checkups.

A metabolic condition with numerous etiologies, diabetes mellitus (DM) is defined by an elevated blood glucose level and abnormalities in the metabolism of carbohydrates, fats, and proteins as a result of problems in insulin secretion, insulin action, or both (2). Insulin resistance is a metabolic disorder known as type 2 diabetes that is characterized by a progressive loss of adequate cell insulin production and a rise in blood sugar levels (hyperglycemia) (1, 3).

Poor eating habits and physical inactivity are the two main modifiable risk factors for developing type 2 diabetes (DM2) in addition to genetic predisposition (4), which accounts for 90% to 95% of all instances of the illness. The increased prevalence of type 2 DM worldwide was influenced by changes in lifestyle and an increase in obesity (5). It is a terrible, widespread chronic condition that frequently results in limb amputations, blindness, renal failure, and stroke (6).

According to the World Health Organization (WHO), increasing the efficacy of self-management support may have a more significant impact on population health than medical therapies. The term “self-management” refers to the alterations in lifestyle required to manage a chronic illness (7).

The study conducted in Debre Markos showed that the majority of respondents, 221 (92.5%), were trying to manage their diet, and only 32.9% of them were trying weight reduction (8).

Self-care is an activities that people take on their own initiative to maintain their own health, happiness, and quality of life. Additionally, it is crucial and absolutely necessary in the overall control of diabetes and is frequently regarded as the cornerstone of diabetic care (9, 10).

Diabetes self-care practices are critical in keeping the disease under control, and as much as 95% of the self-care is usually provided by the patients or their families (11).

Self-management refers to the individual’s capacity to manage the symptoms, treatment, physical and psychosocial significances, and lifestyle changes characteristic of living with a chronic condition (12).

Adhering to diabetic self-care management involves changing one’s lifestyle and may involve taking medication, recommended diet, regular physical activity, foot care practice, and self-monitoring blood glucose (SMBG) (6, 13). Adherence is a potent, complex process with many interrelated components. Factors relating to psychology, and demographics, and society have been linked to diabetes patients’ adherence. Commitment to these self-care practices enhances blood sugar regulation, maintains blood pressure, lessens the severity of problems, and lowers medical expenses (14).

There are various types of diabetes: Type 1 diabetes is an autoimmune disorder where the body’s immune system attacks and destroys the insulin-producing beta cells in the pancreas. This results in little to no insulin production. It is most commonly diagnosed in children, adolescents, or young adults. People with type 1 diabetes are insulin-dependent for life because their bodies cannot produce insulin. In type 2 diabetes, the body either becomes resistant to insulin or does not produce enough insulin. The exact cause is unclear but is often linked to lifestyle factors such as obesity, physical inactivity, and poor diet. Typically develops in adults (over 40 years old), but increasing numbers of children and adolescents are being diagnosed due to rising obesity rates. Insulin is not always required initially, but many people with type 2 DM may eventually need insulin or other medications to control blood glucose.

The significance of self-management compliance in preventing type 2 diabetes complications following self-management guidelines is essential to avoiding type 2 diabetes (T2D) problems. T2D is a chronic illness that can cause blindness, kidney failure, neuropathy, cardiovascular disease, and other long-term health issues if left untreated.

Self-management is crucial for regulating blood glucose levels and reducing the risk of these complications because the disease is chronic. This includes keeping an eye on blood glucose levels, taking medications as directed, eating a healthy diet, exercising frequently, and managing stress. The roles of adherence to self-management in preventing complication are also prevention of long-term complications, control of risk factors for cardiovascular disease, management of comorbidities, prevention of cute complications, psychological benefits, and overall quality of life.

The current study can utilize study populations from different health institutions and have better generalizability. Therefore, the finding of this study would be important for the health professionals, zonal health department, regional and federal ministry of health, and policymakers to emphasize adherence to diabetic self-care management and associated factors among diabetic patients.

Health practitioners can identify as high-risk for disease-related complications early and take prompt action to improve their outcomes by giving education, emphasizing the management of diabetes. Helping patients improve their health and quality of life is considered an important aspect of diabetes self-care for patients and their relatives. Therefore, the findings of the current study will be helpful to design adherence to diabetic self-care practice and to explore different barriers of self-care practice, and also it helps every staff member in the diabetic clinic to improve the health information about self-care practice.

A study in a Gamo Gofa Zone public hospital found that participants who lived in the rural areas were seven times more likely to have poor diabetic self-care than their counterpart (AOR: 7.16; 95% CI 3.31–15.46) (6). A cross-sectional study done in Debre Markos University found that the study participants who were rural residents were 29% (AOR: 0.71, 95% CI 0.40–2.23) less likely to practice self-care habits than those urban residents (8).

## Materials and methods

### Study design, setting, and period

This study employed a concurrent mixed-methods approach in order to offer a more comprehensive understanding of a research problem and was conducted in the North Shewa Zone public hospitals in Ethiopia. For both quantitative and qualitative studies, the data collection was held from May 5 to May 20, 2023. The methods of integration of quantitative and qualitative study were as follows: Sequential integration involves using one method first and then using the other method. The North Shewa Zone is one of the 12 zones found in the Amhara regional state. Its city administration is Debre Berhan, which is located 130 km from Addis Ababa and 695 km from Bahir Dar, the capital city of the Amhara regional state. The total population of the zone is 2,322,148, out of which 1,171,150 are men and 1,150,638 are women. The zone is bordered at the south and the west by the Oromia special zone on the northeast and the Afar region on the east. According to the zonal health department report, the North Shewa Zone has 164 private clinics, 97 governmental health centers, 391 health posts, 8 primary hospitals, 2 general hospitals, and 1 comprehensive specialized hospital. North Shewa Zone has a total of 11 public hospitals (56).

### Study population and inclusion criteria

All type 2 diabetic patients who got services in North Shewa Zone public hospitals were considered as the source population for both quantitative and qualitative study designs. Included were those in this study whose age was greater than 18 years and who had follow-ups for at least 6 months. Clients who could not communicate due to illness and pregnant women were excluded.

### Sample size determination

The sample size required for the study was calculated using Open Epi statistical software, version 3.03. A 95% confidence interval (CI), a 5% margin of error, and a 44.7% prevalence of adherence to diabetic self-care management were the assumptions made. Adding a 10% non-response rate, the estimated sample size was 417 (15). By using a design effect of 1.5, the total sample size becomes 626.

### Sampling procedure

**Figure 1:**
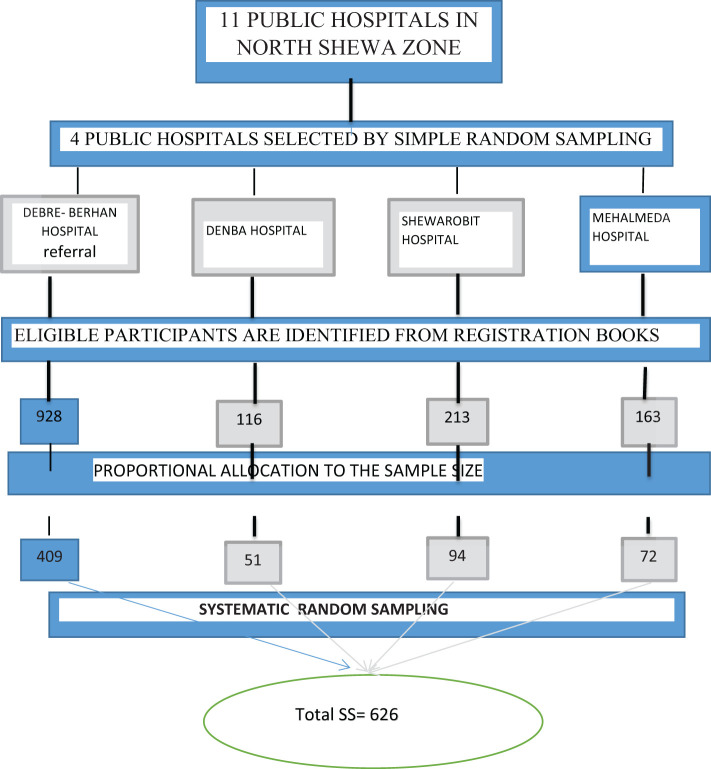
Adherence to diabetic self-care management and it's associated factors among type 2 diabetic patients in North shewa zone public hospitals, Ethiopia 2023.

A simple random sampling technique was used for the selection of hospitals after identifying all public hospitals in the North Shewa Zone. Debere Berhan Comprehensive Specialized Hospital (DBCSH), Mehal Meda General Hospital (MMGH), Deneba Primary Hospital (DPH), and Shewarobit Hospital (SPH) were selected randomly, and then a sample was drawn from each hospital based on primary data from type 2 DM patients who came for follow-up, and their medical record number (MRN) was used as a sampling frame. The total number of T2DM in selected hospitals was 1420 (**Figure 1**). The sample has been allocated proportionally for each hospital. Study participants were selected using a systematic random sampling technique. First, the sampling interval (K) value was determined by dividing the total number of type 2 diabetic patients in the study period by the total sample size, which gives 2. The first participant was chosen by the lottery method, and the rest were chosen at every two intervals until the desired sample size was reached. The number of participants for the qualitative interview was selected by using purposive sampling to select 15 type 2 DM patients from four public hospitals in their specialized diabetic clinic. Participants were selected based on their duration of diabetes monthly follow-up for more than 5 years. From Debre Berhan Comprehensive Specialized Hospital, Deneba Primary Hospital, Mehalmeda General Hospital, and Shewarobit Primary Hospital, participation was using in-depth interviews till the patterns or themes in the data became repetitive and no new information was being revealed through further data collection; the data were theoretically saturated.

### Data collection tool and procedure

Data were collected by using a semi-structured interview-administered questionnaire, which contained five subparts. Part one is sociodemographic factors, which included five questions. Six questions made up the tool’s second section, “Health care facility related factors,” and three questions made up its third section, “Disease-related factors”. The fourth section included two questions and treatment-related parameters. The last one was the outcome variable. Adherence to diabetic self-care activities consisted of five domains, namely, adherence of diet, physical activities, blood sugar testing, foot care, and antidiabetic medication. Each domain has four, two, two, four, and three questions, respectively. Within qualitative tools, age and sex are included in sociodemographic questions for type 2 DM patients, and they also ask about period of diagnosis. Four barriers of adherence to diabetic self-care practice questions were included in the qualitative part. If the data were collected anonymously, confidentiality and beneficence would be assured throughout the study period.

The study participants for both the quantitative and qualitative parts were notified orally and signed an informed consent and were then selected using systematic sampling at every other patient. The first respondent was chosen by lottery from the first two patients to the hospital. A purposive sampling procedure was used to recruit type 2 DM patients for the study based on their sex, age, and the period of their diagnosis (16). This was done to be able to see the different methods of self-care practices from different perceptions.

All in-depth interviews were conducted by the first author in a private space within the hospital compound. The interview was conducted in the Amharic language and tape-recorded with verbal consent obtained from the study participants, and then translated into English. The required data were collected by four BSc nurses. Training was provided for data collectors similar to that of the quantitative part. We used different strategies to mitigate interview and social desirability bias, as follows: standardize interviews, train interviews, minimize non-verbal cues, ensure confidentiality, ensure anonymity, create a comfortable environment, and use indirect questions.

### Study variable

The dependent variable of the study is adherence to self-care. Sociodemographic variables (sex, age, marital status, level of education, occupation/employment), healthcare facility-related factors (access to healthcare facilities, patient satisfaction with quality healthcare services, physician patient relationship, and communication of critical information), disease-related factors (duration of type 2 DM, comorbidity, illness-associated complication), and treatment-related factors (route of administration, duration of treatment) are among the explanatory variables that can be used to explain the data.

### Definition of terms

#### Adherence to diabetic self-care management

✔ Adherence: the extent to which a person’s behavior concerning taking medication, following a diet, and/or executing lifestyle changes corresponds with agreed recommendations from a health provider (9).
✔ Self-care: activities that individuals initiate and perform on their behalf in maintaining life, health, and well-being (9).
✔ Self-care practice: It is a daily regimen task that the individual patients were to perform to manage diabetes on their behalf (dietary practice, exercise, medication, daily foot care, and monitoring blood glucose) (15).
✔ Diabetes self-care practice was assessed by participants’ responses to the 14-item Summary of Diabetes Self-Care Activities (SDSCA) in the last 7 days. Response choices for each question ranged from 0 to 7 based on the number of days on which the indicated behavior was performed (15).
✔ The overall mean score was estimated by the summation of each item of the scale and divided by the total number of questions. Therefore, after calculating the overall mean score, participants who scored equal to or greater than the mean score were classified as having good diabetes self-care practice, and those who scored below the mean were considered as having poor self-care practice (15).

### Data quality assurance

#### Data processing and analysis

Data were cleaned, edited, and entered using EPI data version 4.6 and transported to SPSS version 25 statistical software for further analysis. Descriptive statistics was used to organize and summarize the variables. Bivariable analysis for each independent variable with the outcome variable was performed to select candidates for multivariable analysis. All independent variables with a p-value less than 0.25 were taken as candidates for the multivariable logistic regression model, and then finally p-values of less than 0.05 at 95% CI were used to declare statistical significance. A multicollinearity test was used to see the linear correlation among independent variables by using standard error, and there was no existence of multicollinearity. The Hosmer and Lemeshow goodness-of-fit test was done, and the model was fitted. The adjusted odds ratio (AOR) from multivariable logistic regression was used to measure the strength of association between dependent and independent variables. Finally, summary measures, tables, and figures were used to present the findings.

#### Qualitative part

A thematic method was used to manually examine the data, and as a result, codes and groups that made understanding easier arose according to variable coding steps.

### Data processing and analysis

Data were cleaned, edited, and entered using EPI data version 4.6 and transported to SPSS version 25 statistical software for further analysis. Descriptive statistics was used to organize and summarize the variables. Bivariable analysis for each independent variable with the outcome variable was performed to select candidates for multivariable analysis. All independent variables with a p-value less than 0.25 were taken as candidates for the multivariable logistic regression model, and then p-values of less than 0.05 at 95% CI were used to declare statistical significance. Multicollinearity test was used to see the linear correlation among independent variables by using standard error, and there was no existence of multicollinearity.

The Hosmer and Lemeshow goodness-of-fit test was done, and the model was fitted. The adjusted odds ratio (AOR) from multivariable logistic regression was used to measure the strength of association between dependent and independent variables. Finally, summary measures, tables, and figures were used to present the findings.

#### Qualitative part

A thematic method was used to manually examine the data, and as a result, codes and groups that made understanding easier arose according to the variable coding step.

In addition to the qualitative findings, the findings were provided in textual narrations and quotations, and they supported the quantitative findings using this step. Steps of the thematic method include the following: familiarize yourself the data by reading it multiple times, generate initial codes by tagging significant pieces of data, search for themes by grouping similar codes together, review themes and refine themes, define and name themes, write the analysis, and use data to support the findings.

### Ethical approval

Ethical clearance was obtained from the institutional review board of the Debre Berhan University with institutional research ethics review committee number IRB 01/93/2023. A support letter was written to each hospital. The purpose of the study was clearly explained to the participants, and their written informed consent was obtained before data collection. The study was conducted in accordance with the Declaration of Helsinki ethical principles for medical research on human subjects. This study was conducted after obtaining ethical clearance from the Debre Berhan University College of Health Sciences Ethical Review Board. A formal letter obtained from Asrat Woldeyes Health Science Campus, Debre Berhan University, was submitted to the hospital administration to gain their cooperation. The respondents’ rights and dignity were also respected. Written informed consent was obtained from the study participant to confirm willingness for participation after explaining the objective of the study. The respondents were notified that they have the right to refuse or terminate at any point of the interview. The information provided by each respondent was kept confidential throughout the research process.

## Results

### Sociodemographic characteristics of participants

There were a total of 626 participants, 600 of whom were involved in this study, making a response rate of 96%. Of these, more than half, 323 (53.8%), were men. The age of study participants ranged from 30 to 85, with the mean age of 52.65 years (S.D. ± 12.347). A total of 430 (71.7%) failed within the range of the 30-60-year age group. More than half, 313 (52.2%), of the participants were married. Among the study participants, nearly 371 (61.8%) of the participants were from an urban area. Regarding educational status, 192 (32%) were college and above. In terms of occupation, 445 (74.2%) were unemployed (**Table 1**).

**Table 1:** Sociodemographic characteristics of type 2 diabetic patients in public hospitals of North Shewa Zone, Ethiopia, 2023 (n=600).

Variables	Category	Frequency Percent (%)	
Age in year	30-60	430	71.7%
	61-70	118	19.7%
	>70	52	8.7%
Sex	Male	323	53.8%
	Female	277	46.2%
Marital status	Single/never married	117	19.5%
	Married	313	52.2%
	Divorced	61	10.2%
	Widowed	109	18.2%
Residence	Urban	371	61.8%
	Rural	229	38.2%
Level of education	No formal education	141	23.5%
	Primary School	105	17.5%
Occupation	High School	162	27%
	College and above	192	32%
	Unemployed	445	74.2%
	Employed	155	25.8%

### Healthcare facility-related factors

More than half, 361 (60.2%), type 2 DM participants lived in urban areas. Of the respondents, nearly 350 (58.3%) of them had got healthcare services at any time. According to the findings of this study, around 515 (88.5%) of the participants were satisfied with the quality of healthcare services, and around 525 (87.5%) got sufficient attention from healthcare professionals. Almost 522 (87%) study participants said that “all healthcare practitioners told them about the critical nature of the disease,” but from all study participants, only 245 (40.2%) received published patient education materials on diabetes (**Table 2**).

**Table 2:** Frequency and percentage distribution of healthcare facility-related factors among type 2 DM patients in North Shewa Zone public hospitals, Ethiopia, 2023 (n=600).

Variables	Category	Frequency Percent (%)	
Distance from the hospital.	0-5km	361	60.2%
	6-10km	131	21.8%
	Beyond 10km	108	18%
Availability of healthcare services	Yes	350	58.3%
	No	250	41.7%
Quality of healthcare services	Yes	515	85.8%
	No	85	14.2%
Sufficient attention	Yes	525	87.5%
	No	85	12.5%
Published education materials	Yes	245	40.8%
	No	355	59.2%
Council about the critical nature of self-care	Yes	522	87%
	No	78	13%

#### Disease-related factors

The mean duration of DM was 5.37 years with an SD of ±3.114 years. There were 351 (58.5%) respondents who had DM duration between 1 and 5 years; 111 (18.5%) had DM-related complications. Nearly 148 (24.7%) were admitted to hospitals as a result of diabetes mellitus complications in the last year. With regard to the presence of comorbidity along with diabetes, 358 (59.7%) of respondents had comorbidity, among which hypertension was the most common (173, or 28.8%), followed by heart failure and renal disease (**Table 3**).

**Table 3:** Frequency and percentage distribution of disease-related factors among type 2 DM patients in North Shewa Zone public hospitals, Ethiopia, 2023 (n=600).

Variables	Category	Frequency	Percent (%)
DM duration since its diagnosis, in years	1–5 years 6–10 years >10 years	351 203 46	58.5% 33.8% 7.7%
DM treatment duration, in years	1-5years 6–10 years >10 years	348 203 49	58% 33.8% 8.2%
Comorbidities	Yes	358	59.7%
	No	242	40.3%
Complication	Yes	111	18.5
	No	489	81.5%
History of hospitalization related to DM complication	Yes	148	24.7%
	No	452	75.3%

### Treatment-related factors

**Figure 2:**
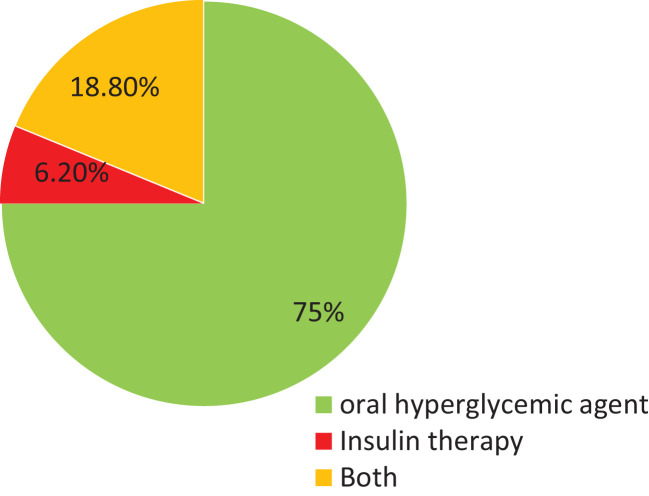
Diabetes management type among type 2 diabetic patients in North shewa Zone public hospitals, Ethiopia 2023.

Most respondents, 450 (75%), took oral hypoglycemic agents. Of all participants, 113 (18.8%) took both an oral antidiabetic drug and insulin for their diabetes management. The remaining study participants were using insulin therapy. Regarding this, 127 (21.2%) took insulin therapy one time per day (**Figure 2**).

### Frequency of adherence to diabetic self-care management

**Figure 3:**
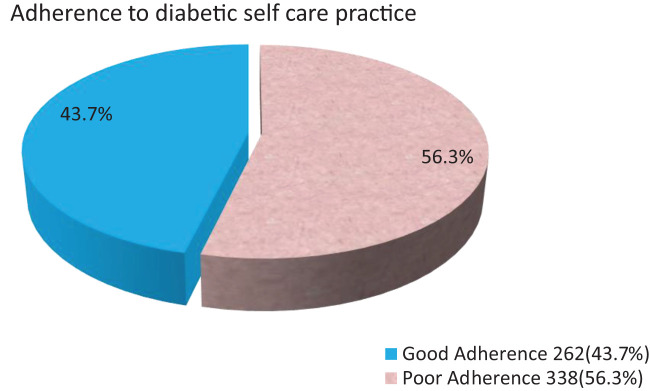
Over all mean adherence to diabetic self care practice among type 2 diabetic melletius patient's in North shewa Zone public Hospitals, Ethiopia 2023.

The majority 91.8% of the study participants had the self-care practice of taking recommended medication, nearly 41% of respondents had the self-care practice of regular physical activity for over 30 min more than 3 days per week, and 184 (30%) of respondents reported they checked their feet every day. Nearly 35.8% of respondents had good diet adherence, and also around 26% of the respondents had good blood glucose tests. The overall mean score for self-care among the study participants was 1.4367 (SD ±0.49639). Overall, 262 (43.7%) of participants had good self-care practices (**Figure 3**).

### Adherence to diabetes self-care and its barriers

A total of 15 type 2 DM patients participated in the qualitative interview study. The participants’ ages ranged from 35 to 68 years; with regard to their sex, nine participants were men, whereas the remaining were women. Their duration of diagnosis ranged from 1 to 12 years. The six themes related to barriers for adherence to diabetic self-care practice that were recognized were lack of education, financial affordability, limited awareness, and negligence toward adherence to diabetic self-care practices, accessibility, lack of social support, and having diabetic-related complications.

#### Theme 1: Negligence

Most of the respondents consider their anti-diabetes medications as the most important component of diabetes management and their existence, but they should not omit appropriate doses.

*“…I don’t take my medicine on time*….” (65-year-old female respondent)

#### Theme 2: Limited awareness and negligence toward diabetic self-care practices

Diabetic patients were not appropriately aware of the importance of diet and did not know how to prepare their meals or family meal preparation habits. Most of the respondents reported being properly counseled by their physicians to do regular exercise and its importance also and about uses of foot care in their diabetes self-care.

“*Health care providers guided me to do easy exercise at least for 30 minutes per day to take care of foot cleanliness. But I have done it irregularly as I needed, and I washed my foot at bedtime.*” (38-year-old male patient)

“*Frankly speaking, I live nearest to the hospital, but I will not come to the hospital until my condition is predominantly serious. Even though healthcare providers taught me about diabetic self-care, I didn’t give attention to myself till now.*” (57-year-old male respondent)

#### Theme 3: Lack of family support

Lack of support in the family setting can create difficulties for diabetic patients when handling the illness in everyday life and practicing adherence to diabetic self-care. All participants revealed that social support had a great role in coping with stressful events. On the other hand, those who compared themselves with other patients needed to undergo medical checkups more frequently. Due to this reason, family support contributed to their own role, such as following treatment recommendations, attending clinic appointments, and supporting other diabetic self-care practices. This result showed that most participants had no family support as needed.

“*My husband died not too long ago, and my kids are too young. So I don’t really take care of myself, and also I don’t have anyone to take care of me.*” (39-year-old female respondent)

**Factors associated with adherence to diabetic self-care management**

Based on bivariable analysis, in the study participants aged between 61 and 70 years (COR 0.68, 95% CI), 32% were more likely to have good adherence than those aged between 30 and 60 years. Those over 70 years (0.16, 95% CI (0.07-0.37)) were less likely to have good adherence than those aged between 30 and 60 years. Regarding gender, women (COR: 1.2 times more likely) had good adherence than men. Regarding the duration of type 2 DM diagnosis, 1–5 years (COR 5.5-CI (2.5-12.1) were 5.47 times more likely to have good adherence than the >10-year duration; similarly, the duration of type 2 DM diagnosis of 6–10 years (COR 2.3-CI (1.01-5.2)) was 2.3 times more likely to have good adherence than the >10-year duration of type 2 DM diagnosis. Those who have medical complications related to diabetes (COR 0.26-CI (0.2-0.4)) were 74% less likely to have good adherence than those who have no medical complications related to diabetes; this is also supported by qualitative methods as below.

#### Theme 4: Diabetic-related complications

Almost all participants reported that this diabetic disease was an illness that dictated changes in lifestyle and living habits. It was essential to modify the diet and to increase physical activity. The most commonly mentioned reasons for not doing regular physical exercise were feelings of pain or disturbed daily activities due to DM-related complications, lack of interest, and lack of motivation.

“*Of course! In case of diabetes, my vision is blurred and disturbed to daily activities; for instance, I stopped getting out of the house as usual; also, I was forced to leave my business.*” (55-year-old male respondent)

“*I know diabetes is incurable and has its own complications because I lived with diabetes for a long period of time, and it resulted in a kidney problem and hypertension. I felt weak and in pain, so I am not interested in doing different activities.*” (52-year old female respondent).

However, in multivariable analysis, living place, level of education, occupation, and availability of healthcare services at any time were significantly associated with adherence to diabetic self-care practice to be a candidate for multivariable logistic regression analysis. Based on this finding, people with type 2 diabetes who lived in urban areas were approximately [AOR-5.5, CI (1.1-8.8)] five times more likely to have better adherence to diabetic self-care practice than rural people.

#### Theme 5: Accessibility (distance)

Most of the respondents lived in the rural area; their appointment time is passing due to lack of accessibility of hospitals and clinics around them. The majority of the respondents reported that they do not regularly check their blood glucose level because they live in distant areas. It indicates a high risk for diabetic complications due to poor glycemic control.

“*…I live in a rural area and could not get to nearby hospitals and clinics….”* (48-year-old female respondent) “*I prefer more frequent checkups. When I know that the doctor is going to see the results of my blood glucose level. I am more careful about the food I eat and the increment of blood glucose by measuring it….*” (42-year-old male respondent)

The respondents who were attended to at the high school level [AOR: 2.98, CI (1.3-6.7)] were 2.98 times more likely to have good adherence to diabetic self-care practice, and also those who were attended to at a higher level of education, college and above [AOR: 5.1, CI (2.1-12.5)], were five times more likely to have a statistically significant association with their adherence condition. This result is also supported by the following qualitative results.

#### Theme 6: Lack of information

Some participants are unable to read and write; some of them have a lack of information due to healthcare providers not being well informed about adherence to diabetic self-care practice during follow-up.

“*My children are not around all the time, and I’m not well educated, so I don’t know all the things I should do to take care of myself and miss out on my medication.*” (54-year-old female respondent)

The study participants who are unemployed [AOR: 0.34, CI (0.15-0.77)] were less likely by 66% to adhere to diabetic self-care practice than those who are employed. From the total respondents who had no availability of healthcare services at any time [AOR: 0.19, CI (0.09-0.37)], there was less likelihood by 81% of adherence to diabetic self-care practice than availability of healthcare services at any time (**Table 4**). This result is also supported by the following qualitative results.

**Table 4:** Bivariable and multivariable logistic regression analysis on study of adherence to diabetic self-care management and associated factors among type 2 diabetic patients in North Shewa Zone public hospitals, Ethiopia, 2023 (n =600).

Variables	Adherence to self-care practice		COR CI 95%	AOR CI 95%	p-value
	Good	Poor			
Age					
30–60 years	209 (34.8%)	221 (36.8%)	1	1	0.124
61-70 years	46 (7.7%)	72 (12%)	0.68 (0.45-1.02)	1.54 (0.82-2.89)	0.182
>70 years	7 (1.2%)	45 (7.5%)	0.16 (0.07-0.37)	0.51 (0.19-1.38)	0.184
Gender					
Male	134 (22.3%)	189 (31.5%)	1	1	
Female	128 (21.3%)	149 (24.8%)	1.21 (0.88-1.68)	1.53 (0.99-2.36)	0.058
Residence					
Urban	221 (36.8%)	151 (25.2%)	6.67 (4.49-9.92)	5.49 (1.05-8.78)	0.044*
Rural	41 (6.8%)	187 (31.2%)	1	1	
Level of education					
No formal education	18 (3%)	123 (20.5%)	1	1	
Primary school	10 (1.7%)	95 (15.8%)	0.71 (0.32-1.63)	0.41 (0.16-1.08)	0. 070
High school	93 (15.5%)	69 (11.5%)	9.21 (5.13-16.52)	2.97 (1.33-6.67)	0.008*
College and above	262 (43.7%)	338 (56.3%)	18.9 (10.5-34.1)	5.08 (2,07-12.5)	0.000*
Occupation					
Unemployed	146 (24.3%)	299 (49.8%)	0.16 (0.11-0.25)	0.34 (0.15-0.77)	0.010*
Employed	116 (19.3%)	39 (6.5%)	1	1	
Availability of healthcare services					
Yes	234 (39%)	116 (19.3%)	1	1	
No	28 (4.7%)	222 (37%)	0.06 (0.04-0.09)	0.19 (0.09-0.37)	0.000*
Duration of type 2 DM diagnosis					
1–5 years	188 (31.3%)	163 (27.2%)	5.48 (2.49-12.08)	9.26 (0.80-10.7)	0.075
6–10 years	66 (11%)	137 (22,8%)	2.29 (1.01-5.18)	3.34 (0.22-5.51)	0.055
>10 years	8 (1.3%)	38 (6.3%)	1	1	
Medical complication related to diabetes					
Yes	22 (3.7%)	89 (14.8%)	0.27 (0.16-0.42)	0.56 (0.15-2.06	0.380
No	240 (40%)	249 (41.5%)	1	1	

*p-value <0.25 in bivariable analysis, ***p-value <0.001, **p-value <0.01, *p-value <0.05 in multivariable analysis and 1 indicates the reference variable. COR, crude odd ratio; AOR, adjusted odd ratio; CI, confidence interval.

#### Theme 7: Financial affordability

Most study participants felt that living with DM was very expensive, which required them to make adjustments in many things. They recognized diet as a vital component of self-care practice for people living with diabetes. Affordability of healthy food is a commonly mentioned barrier to accepting a recommended diet strategy. In this study, respondents could not afford to buy appropriate and recommended food since most of them were unemployed.

“*…I really try to take care of myself in every way, but it’s hard to keep up with this current living condition….*” (35-year-old male respondent).

## Discussion

The magnitude of overall good adherence to diabetes self-care practice was 262 (43.7%) with (95% CI: 40–47.8%) among type 2 DM diabetic patients in North Shewa Zone public hospitals. This study was consistent with the study conducted in Debre Berhan Referral Hospital in Northeast Ethiopia (44.7%) (15), Debre Markos (48.5%) (8), Hawassa University Comprehensive Specialized Hospital (47.8%) (9), Hossana, southern Ethiopia (43.1% hyperglycemia) (2, 3), and the Philippines (43.7%) (17).

However, the finding of this study was greater than the study conducted in Arba Minch General Hospital, Southern Ethiopia (41.2%) (18), and Tigray Region, Ethiopia (37.3%) (19).

This discrepancy may be due to some enhancements in the healthcare services related to the period gap.

On the contrary, the finding of this study was lower than the study conducted in Dessie Referral Hospital, northeastern Ethiopia (55.3%) (16); Dire Dawa (55.9%) (20); Eastern Ethiopia Harer (53.2%) (21); Hadiya Zone (52.3%); Dilla University, South Ethiopia (76.7%) (22); Gamo Gofa Zone Public Hospital (53.7%) (6); and Morocco (66.6%) (3, 23). Of the study participants, 50.3% had good self-care practices.

This variation could be due to sociocultural differences, different study periods, and a study area that means that the North Shewa Zone is a very wide area and the population lives in rural areas more scattered than other cities; it might be a reason for health service utilization, the educational status of the respondent, and the current economic crisis, which can be some of the major justifications for the lower result of good adherence to diabetic self-care practice.

This study revealed that those who lived in urban areas were 5.4 times more likely to have good diabetic self-care practice than those rural residents. This is consistent with a study conducted in Debre Markos (8) and Gamo Gofa Zone public hospital (6).

In the present study, the respondents who had a high school level were 2.9 times more likely to be engaged in good self-care practice, and those who had college and above were five times more likely to have good self-care practice when compared with respondents who had no formal education. This finding was congruent with the studies conducted in Dire Dawa (20), Addis Ababa (11), and Ghana (24). This indicates that education is the base for a diabetic patient to understand the disease process and to provide their own self-care practice because they may be able to read and become well-informed of the benefits of adherence.

Based on the study, unemployed people were less likely by 66% to have good self-care practices than employed people; this finding was in line with the study conducted in Addis Abeba (11) in Debre Markos (8), and the study conducted in Tigray region, Ethiopia (19). Respondents who had no availability of healthcare services at any time were 81% less likely to adhere to diabetic self-care practices than those who had availability of healthcare services at any time. This finding was congruent with a study conducted in a Kenya referral hospital (25).

A complete self-care practice among type 2 diabetic patients was uncommon. Most of the respondents entirely depended on their medications, feeding habits, and measuring blood glucose levels to manage their illnesses and its complications and tended to undermine the importance of the other elements of self-care either due to lack of education, financial affordability, limited awareness, and negligence toward adherence to diabetic self-care practice, accessibility, or lack of social support.

In this study, financial affordability was the major barrier to good adherence to diabetic self-care practice elements. This finding was in line with the study conducted in Addis Ababa, Ethiopia (26). Similarly, the study done in Tigray, Ethiopia, indicated that the too restrictive nature of diet recommendations and financial problems had a negative influence on their adherence to self-care behaviors (27).

The findings from the study revealed that the majority of the respondents had a lack of education, limited awareness, and negligence toward adherence to diabetic self-care practices; some of the negligent respondents had gotten access, but they should not give attention to themselves toward self-care practices. This finding was congruent with the studies conducted in Addis Ababa, which revealed that some of the participants also reported that adhering strictly to diabetes dietary recommendations is boring and practically impossible (26) and that education from physicians is one of the most important determinants for change in self-care practices (4).

On the other hand, this study explored patient compliance; the respondents feel hopelessness due to the disease being incurable, and they preferred alcohol to the recommended drug, and some of them faced loss of social support. This finding is in line with the study conducted in Ghana. Most patients reported that these family members assisted with a wide range of management strategies, such as adjusting to new diet regimes and roles dictated by the patient and assisting with the preparation of recommended diet (28). A similar study in Pakistan found that getting support from family members is one of the important determinants of compliance with medication-taking behavior (4).

In case of diabetic-related complications, these study participants were disturbed to perform daily activities, unable to do their business, and faced an economic crisis. This finding was in line with the study conducted in Dessie Referral Hospital that low-risk perception of complications among DM patients may make them reluctant to practice recommended self-care (16). A study conducted in Singapore showed that participants expressed an awareness of risking diabetes-related complications due to poorly controlled diabetes (29).

## Limitations of the study

Self-care practices are determined based on the respondents’ self-reported values; performance of these activities was not observed and could not be confirmed. Interviewer bias and social participants may provide socially desirable answers about their adherence to self-care management, especially when discussing sensitive topics such as medication adherence or diet, which can skew the results, which were another limitation for this study.

## Conclusion and recommendation

This study indicated that adherence of patients with type 2 diabetes to the recommended self-care practices was considerably poor. Different factors, including the respondents who have a high school level or higher levels of education, people with type 2 diabetes who lived in urban areas, the respondents who were unemployed, and people with type 2 diabetes who had no availability of healthcare services at any time, had statistically significant associations with adherence to diabetic self-care practice according to the quantitative study. The findings suggest that sociodemographic factors such as age, education level, and economic status play a significant role in influencing self-care behaviors. Older patients, those with higher education levels, and those with better financial stability tend to show better adherence to prescribed self-care routines, including medication and diet. On the other hand, patients from lower socioeconomic backgrounds face challenges in accessing medication, attending regular medical appointments, and acquiring the necessary resources for maintaining a healthy lifestyle. This was supported by the results from the qualitative part and thus the endorsement to strengthen diabetes health education to patients and their relatives.

✔ We recommend that policymakers should have to develop health information dissemination programs and strategies to improve the awareness of diabetic patients about the importance of diabetic self-care practices.
✔ Regional Health Bureau and Zonal Health Department with North Shewa Zone public hospitals diabetic clinic coordinators should give greater emphasis toward accessibility of nearby health facilities around the rural area as much as possible. Healthcare professionals should counsel and educate DM patients during follow-up and need to spend more time alerting patients about diabetic self-care.
✔ Providing short-term training for healthcare providers in hospitals to improve patient education on diabetes management.
✔ Medium-term: development of community-based educational programs that involve family support.
✔ Long-term: integration of diabetes adherence programs into Ethiopia’s primary healthcare system.
✔ Other researchers are better to explore unanswered issue**s** toward factors affecting adherence to diabetic self-care management.

## Data Availability

The datasets presented in this study can be found in online repositories. The names of the repository/repositories and accession number(s) can be found in the article/supplementary material.
